# COIL: a methodology for evaluating malarial complexity of infection using likelihood from single nucleotide polymorphism data

**DOI:** 10.1186/1475-2875-14-4

**Published:** 2015-01-19

**Authors:** Kevin Galinsky, Clarissa Valim, Arielle Salmier, Benoit de Thoisy, Lise Musset, Eric Legrand, Aubrey Faust, Mary Lynn Baniecki, Daouda Ndiaye, Rachel F Daniels, Daniel L Hartl, Pardis C Sabeti, Dyann F Wirth, Sarah K Volkman, Daniel E Neafsey

**Affiliations:** Department of Biostatistics, Harvard School of Public Health, Boston, MA 02115 USA; Department of Immunology and Infectious Disease, Harvard School of Public Health, Boston, MA 02115 USA; Laboratoire de Parasitologie, National Reference Centre for Malaria, Institut Pasteur de la Guyane, Cayenne, French Guiana, France; Faculty of Arts and Sciences, Harvard University, Cambridge, MA 02138 USA; Broad Institute of MIT and Harvard, Cambridge, MA 02142 USA; Faculty of Medicine and Pharmacy, Cheikh Anta Diop University, Dakar, Senegal; Simmons College School of Nursing and Health Sciences, Boston, MA 02115 USA

**Keywords:** Malaria, *Plasmodium*, *vivax*, *falciparum*, Complexity of infection, Multiplicity of infection, SNP, Barcode, Genotype, Likelihood

## Abstract

**Background:**

Complex malaria infections are defined as those containing more than one genetically distinct lineage of *Plasmodium* parasite. Complexity of infection (COI) is a useful parameter to estimate from patient blood samples because it is associated with clinical outcome, epidemiology and disease transmission rate. This manuscript describes a method for estimating COI using likelihood, called COIL, from a panel of bi-allelic genotyping assays.

**Methods:**

COIL assumes that distinct parasite lineages in complex infections are unrelated and that genotyped loci do not exhibit significant linkage disequilibrium. Using the population minor allele frequency (MAF) of the genotyped loci, COIL uses the binomial distribution to estimate the likelihood of a COI level given the prevalence of observed monomorphic or polymorphic genotypes within each sample.

**Results:**

COIL reliably estimates COI up to a level of three or five with at least 24 or 96 unlinked genotyped loci, respectively, as determined by *in silico* simulation and empirical validation. Evaluation of COI levels greater than five in patient samples may require a very large collection of genotype data, making sequencing a more cost-effective approach for evaluating COI under conditions when disease transmission is extremely high. Performance of the method is positively correlated with the MAF of the genotyped loci. COI estimates from existing SNP genotype datasets create a more detailed portrait of disease than analyses based simply on the number of polymorphic genotypes observed within samples.

**Conclusions:**

The capacity to reliably estimate COI from a genome-wide panel of SNP genotypes provides a potentially more accurate alternative to methods relying on PCR amplification of a small number of loci for estimating COI. This approach will also increase the number of applications of SNP genotype data, providing additional motivation to employ SNP barcodes for studies of disease epidemiology or control measure efficacy. The COIL program is available for download from GitHub, and users may also upload their SNP genotype data to a web interface for simple and efficient determination of sample COI.

**Electronic supplementary material:**

The online version of this article (doi:10.1186/1475-2875-14-4) contains supplementary material, which is available to authorized users.

## Background

Complexity of infection (COI) in the context of malaria may be defined as the number of genetically distinct *Plasmodium* parasite types simultaneously infecting a patient. Complex (COI >1) infections may be generated by multiple bites from parasite-infected mosquitoes or by a single bite from a mosquito infected with multiple genetic parasite types. COI is an important parameter to measure for two reasons: 1) its potential bearing on clinical disease outcome and, 2) the information it can provide about disease transmission.

The relevance of COI to disease manifestation and immune response is well established, but mechanisms are not yet understood and correlations are sometimes contradictory. Investigators have found an inverse association between COI and patient age in diverse geographic study sites [3–6], suggesting that acquired immunity to specific parasite strains may cumulatively impact COI. Immunization with the SPf66 [7] or RTS,S/AS02A [8] vaccines has been found to reduce both COI and disease morbidity, suggesting a possible causal link between these two phenomena. While some studies have documented a positive association between COI and symptomatic malaria [9, 10] others have found an inverse association [11–13], suggesting that the clinical and epidemiological consequences of COI are important but not yet fully understood. One recent study found a highly significant positive association between COI and severe malaria in Kampala, Uganda [14]. The true relationship between COI and disease outcome may differ in *Plasmodium vivax* from *Plasmodium falciparum*, and disease outcome may further be complicated by simultaneous infections of both species.

Knowing the distribution of COI across a patient population can reveal much about the nature of disease transmission and epidemiology. For *P. falciparum* infections, COI in Senegal has been found to be seasonal and positively associated with the degree of parasitaemia in individual patients [15]. In Sudan and Tanzania, COI has been used as a metric to study the rate of disease transmission [16, 17]. Nkhoma and colleagues recently observed that polymorphic SNP genotypes (expected if COI >1) declined in prevalence over the course of a decade along the Thai-Burma border, in tandem with a decline in disease transmission in that region [18].

Given the usefulness of COI, a variety of methods have been developed for estimation of this statistic from clinical samples. The most common method to evaluate COI involves PCR amplification of one or more genetic loci (*msp1*, *msp3*, *glurp*) known to be segregating numerous insertion/deletion (indel) polymorphisms, and then estimating COI as the maximum number of amplicon-length alleles observed at the most diverse locus for a given sample [19]. This method requires subjective interpretation of agarose gels, has been observed to yield inconsistent PCR results, and may be insensitive due to the relatively small number of indel polymorphisms surveyed [20]. Other methods of evaluating COI have focused on reconstructing haplotypes from sequencing or genotyping data at one or a few loci [21]. These haplotype-reconstruction approaches are computationally intensive, making their application to more than five loci hard to implement, with consequent limited sensitivity for reliable COI estimation in highly complex infections. The *F*_*WS*_ metric of Auburn and colleagues [22] captures within-host heterozygosity relative to population diversity from sequence-based SNP data, but does not provide a direct estimate of COI. The estMOI software developed recently by Assefa and colleagues [23] utilizes phasing information of alleles in SNP-dense regions of the genome to estimate COI, but requires deep whole genome shotgun sequence data, which is costly to generate at large scale.

With the advent of sequence-based genome-wide polymorphism catalogues for many parasite populations, it is becoming feasible to design panels of SNP-based genotyping assays for assessing parasite genetic diversity and geographic origin in collections of clinical samples [18, 24–28]. Because *Plasmodium* parasites are haploid during the stages of their life history during which they infect humans, the presence of polymorphic genotype calls in a SNP-based genotyping assay panel, or molecular barcode, is a clear indication that COI is greater than one for a particular sample. However, no formal method exists to directly estimate COI from genotyping data in a haploid organism such as *Plasmodium spp*.

Given the potential importance of COI and the potential inaccuracy or computational complexity of existing methods for estimating COI, there was a perceived need for new tools. This manuscript describes a computationally efficient, likelihood-based method that produces an estimation of COI from SNP genotyping data, using binomial probability calculations of monomorphic *vs* polymorphic genotype likelihoods produced using the population minor allele frequency (MAF) of the SNPs comprising the barcode panel. Via *in silico* simulation and empirical validation, it is shown that SNP barcodes containing at least 24 unlinked assays can reliably resolve COI up to a level of three, and that panels of at least 96 unlinked assays can resolve COI up to a level of five, which is sufficient for a majority of transmission conditions. Though this manuscript focuses on applications to *Plasmodium spp*., this method is suitable for application to any haploid organism with polymorphism genotype data meeting the assumptions described below.

## Methods

COIL employs a Bayesian approach to assign probabilities to distinct COI levels (*C*) given the data in a genotyping barcode (*G*). This approach requires a prior probability distribution for COI and the ability to compute the likelihood of COI given a barcode, which is equivalent to computing the probability of observing the barcode at a given COI:


Two assumptions are made to simplify the computation of the COI likelihood. The first is that the individual assays in the barcode (*G*_*i*_) are independent, and do not exhibit significant linkage disequilibrium (LD) with other assays. In most *P. falciparum* and *P. vivax* populations, LD is found over physical distances of 10 kb or less [29, 30], suggesting a minimum spacing for SNP assays used in this application. When this assumption of assay independence is met, it means that the probability of observing the composite barcode genotype for a sample with a particular COI is equivalent to the product of the probability of observing the results of each of the component assays:


The second simplifying assumption is that the individual parasite lineages comprising a complex infection are independent and randomly selected from the larger parasite population. In a complex infection assayed for biallelic polymorphisms, the contribution of each strain to the final genotype for each particular assay is therefore equivalent to a series of independent Bernoulli trials, and the overall tally of the number of alternate (non-wild-type/non-reference) alleles conditional upon a given COI follows a binomial distribution. If one denotes *X*_*i*_ as the count of alternate alleles at site *i* with minor allele frequency, then


Biallelic genotyping assays yield three possible results (not including assay failure) for a haploid pathogen like *Plasmodium*, enumerated as:

0. No alternate alleles (monomorphic reference)
1. Only alternate alleles (monomorphic alternate)
2. Mixture of reference and alternate alleles (polymorphic)

The probabilities of observing these outcomes can be computed using the binomial distribution:


Assay failures are ignored and do not contribute to the likelihood calculation. The minor allele frequencies for each assay can be supplied if they are already known for a given population. Alternatively, they can be estimated from singleton infections (which are assumed to be those containing no polymorphic calls) in a given sample. For the methods here, a Bayesian approach to assay MAF is used, where the allele counts are expected to follow a binomial distribution and a *Beta*(1,1) prior distribution is applied to the MAFs (which is equivalent to the *Uniform*(0,1) prior). The effect of doing this is to pad the counts of the alleles by 1, which is useful when the minor allele frequency is small and the minor allele is not observed in singleton infections.

This approach was further refined by building in a tolerance for inevitable genotyping errors. A genotyping error is defined as a scenario in which the observed outcome of an assay (*G*_i_^*^) does not match the true state of the polymorphism in the sample. The error rates (**E**) can be described as a stochastic matrix as follows:


Using this matrix, one can calculate the probability of observing any outcome of the assay as:


To find the posterior COI distribution, the likelihood for each COI level is multiplied by the prior probability for that COI, and then the likelihoods are normalized to sum to one. The choice of prior can be informative if there is some information about the prevalence of complex infections. Alternatively, the COI prior can simply be defined as a discrete uniform distribution with the maximum value being the limit of detection for the given barcode. The default prior on the web implementation of COIL assumes no external information exists about the prevalence of complex infections, and utilizes a uniform distribution with a maximum COI level of five. The downloadable version of COIL allows users to manipulate the prior.

## Results

**Figure 1:**
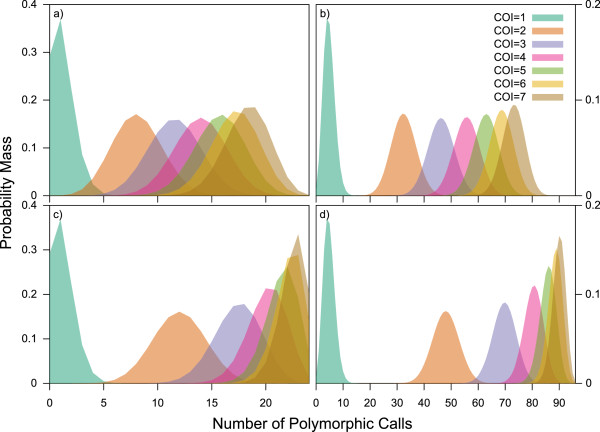
***In silico*** **analysis of resolution.** Analyses of probability mass distributions for COI 1-6 using **a)** 24 SNPs with MAF = 0.2, **b)** 96 SNPs with MAF = 0.2, **c)** 24 SNPs with MAF = 0.4, and **d)** 96 SNPs with MAF = 0.4. Separation of distributions is positively associated with resolution power of the COIL algorithm.

The fundamental utility of this algorithm derives from the property that, for a given minor allele frequency and genotyping error rate, the number of polymorphic calls in a composite genotype is expected to follow a binomial distribution, the moments of which are dependent on the underlying COI of the sample. Figure 1 illustrates the degree to which the underlying probability mass distributions of the expected number of polymorphic calls vary depending on MAF, for barcodes based on 24 *vs* 96 assays. Increasing the MAF from 0.2 to 0.4, or the number of assays from 24 to 96, greatly increases the separation of the probability mass distributions for samples of different COI levels, especially when COI is less than five. Mass distributions for samples with COI = 1 depart from the y-axis (zero polymorphic calls) due to the built-in tolerance for genotyping errors. The power of this algorithm is contingent on the separation of these probability mass distributions within the relevant COI range.

**Figure 2:**
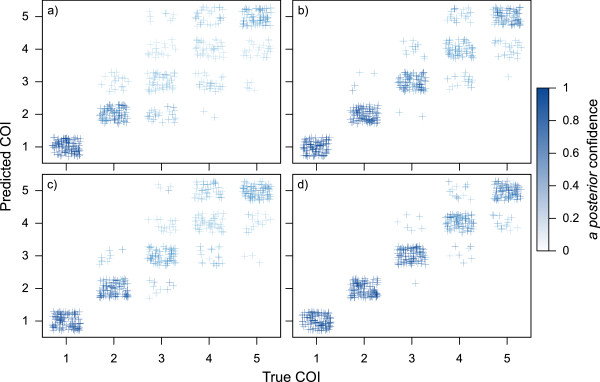
**Validation with simulated genotype data.** Plots represent algorithm performance for *in silico* simulated samples exhibiting "true" COI levels between 1 and 5 (100 samples per COI level), with varying grades of genotyping data: **a)** 24 SNPs with MAF = 0.2, **b)** 96 SNPs with MAF = 0.2, **c)** 24 SNPs with MAF = 0.4, and **d)** 96 SNPs with MAF = 0.4. Accuracy improves with more SNPs and higher MAF.

The potential performance of this approach was first evaluated using *in silico* simulations. Simulated genotypes were generated for samples with a true COI ranging from one to five, under conditions in which the assayed SNPs all had minor allele frequencies of 0.2 or 0.4, and in which the size of the assay set was either 24 or 96. After generating 100 simulated genotypes for each condition under a range of COI levels, the genotypes were run through the COIL algorithm and checked for concordance between the true COI and the maximum *a posteriori* COI predicted by COIL. Figure 2 illustrates the outcome of this *in silico* experiment. Accuracy is relatively low with 24 assays and an assay MAF of 0.2 (Figure 2a), but improves markedly up to a level of COI = 3 (84.4% correct calls) when assay number is kept the same and MAF is increased to 0.4 (Figure 2b). For a barcode of 96 assays, performance is improved at MAF = 0.2 (Figure 2c) relative to the set of 24 assays, but remains deficient for COI >3. For a barcode containing 96 SNPs with MAF = 0.4 (Figure 2d), accuracy is high up to a COI level of five (91.4% correct calls). These results indicate that while increasing assay number can compensate somewhat for low assay MAF, high assay MAF is a very important parameter for reliable COI estimation at complexity levels greater than two.

Next, the performance of the COIL algorithm was evaluated on a collection of empirical genotype data from 21 clinical DNA samples obtained from patients infected with *P. vivax* in French Guiana. *Plasmodium vivax* infections have been increasing in prevalence in this region over the past ten years. *Plasmodium vivax* is distinct from *P. falciparum* in its capacity to remain latent in the liver and induce relapse infections weeks or months later, leading to the expectation that polygenomic infections could be more common even in relatively low transmission settings. Each sample was genotyped at 36 SNPs using an Illumina Eco platform, as described elsewhere [31]. Each sample was also previously genotyped for length polymorphisms at 15 microsatellite loci (Additional file 1), and COIL was run using previously determined MAF estimates for each assayed SNP (Additional file 2). Because microsatellite loci commonly harbour more than two segregating alleles, sample COI was evaluated as the maximum number of alleles observed at any single locus within the microsatellite panel. The microsatellite-based COI estimates and the SNP-based COI estimates produced with COIL were found to exhibit general concordance (Table 1).

**Table 1:** **Concordance of SNP and microsatelitte**-**based COI estimates in 21 clinical** ***Plasmodium vivax*** **samples**

Sample	SNP barcode	No. poly SNPs	SNP estimated COI [95% CI]	Max. posterior probability	MS estimated COI	No. poly MS	SNP/ MS agree?
L066	CGTCGTACAGACCCATCCATCCGCTGCACCACGTAA	0	1 [1,1]	1	1	0	Yes
L099	CGCTNCACNAACCTATCCAGNCANTGNNNCNAGTAC	8	2 [2,2]	0.9994	2	2	Yes
N121	CGTCGCGCGGACCTGTCCGTCCGCTACGCTGAACGC	0	1 [1,1]	1	1	0	Yes
N188	CGCCGTACGGACTTGTCCGTTCGTTGCGCTGAATAC	0	1 [1,1]	1	1	0	Yes
N257	NGNNNNANNNNCTCANNNNGNNNCNNNGCCAAGNNC	21	3 [3,5]	0.5813	3	7	Yes
N315	CGNNNNGTAAACNCATCCNGNCGCTACGCNNAGNAN	11	2 [2,2]	0.9881	2	2	Yes
N426	CGNTNNATNNNCNCATNNNNCTNNNNCGNNNAGCAN	19	3 [2,4]	0.6174	3	6	Yes
N464	CGTCGCACGGACTCACCTGTTCGCTGCGCCGAGCAC	0	1 [1,1]	1	1	0	Yes
N471	CTCCGTACGGACTTGTCCGTCCGCTGCGCCAAGTAC	0	1 [1,1]	1	1	0	Yes
M396	CNCCGTNCGGACCCANNCANCCNCTNCGCCNNGNNN	12	2 [2,2]	0.972	2	1	Yes
J073	CGCTGCATNNACCTGNCNNGTCNCNNCGCCAAGTAC	8	2 [2,2]	0.9989	2	3	Yes
J116	CGTCATGTAGGCCTACCTGGCCGCTGCGTCACGTAC	0	1 [1,1]	1	1	0	Yes
J244	CGTTATATGGACCTACCCAGCTGCCGCGCCAAATAC	0	1 [1,1]	1	1	0	Yes
J255	CGCCGTGCGGACTTGCCCGTTCATTGCGCCAAGTAC	0	1 [1,1]	1	1	0	Yes
J268	CGCTATACAAACCCATCCGTTCACTGTGCCACGCAC	0	1 [1,1]	1	1	0	Yes
L285	CGCCGTGCNGACTNGTCCGTCCGCTGCNCCAAGCNC	4	1 [1,2]	0.7197	2	3	Partial
J096	NGCNGNACGGACTTGCCCGTCCGTTGCGCTGAATAC	3	1 [1,1]	0.9988	2	2	No
L110	CNCCGTACNNANTTNNCCGTCNGTTGNGCTGAATAC	8	2 [2,2]	0.999	1	0	No
N427	CGCTATACGAACTTACCTATCTGCTGCGCCAAGCGC	0	1 [1,1]	1	2	1	No
N492	CGTCGCACAAGCTCGTCCGGTCGTTGCGCTGAGTAC	0	1 [1,1]	1	2	1	No
N524	CGNCGTNTNAACCCNCCNGGCTNCCANNCTAAGNNC	10	2 [2,2]	0.9989	3	5	No
M496	CGCTGCATAAACCCATCTAGTTACTGCGCCACGTAC	0	1 [1,1]	1	2	1	No
J249	CTCCGCACAAACTCGTCCGTCTACTACGCCGAGCAC	0	1 [1,1]	1	2	2	No

In 15 of 23 samples there was perfect agreement between the maximum *a posteriori* COIL estimate of COI and the maximum number of alleles observed at any microsatellite locus. In one case (sample L285) the maximum *a posteriori* COIL estimate was incorrect, but the 95% credibility interval captured the microsatellite-based COI estimate. The remaining seven cases of disagreement in COI estimates fall into three categories. In some cases COIL may underestimate COI as a result of over-tolerance for occasional SNP genotyping errors that render polymorphic calls from presumably monomorphic loci (e.g., sample N524, which exhibits ten polymorphic calls but is called as COI = 2 by COIL). In other cases, COIL may be correctly estimating COI, but the microsatellite data may be incorrectly indicating a higher COI level on the basis of a single locus yielding two or more alleles with similar lengths (Additional file 1; e.g., samples N427, N492). Resolution of two microsatellite alleles with a length difference of 10 bp or less could be subject to interpretation error, and the biallelic microsatellite results for samples N427 and N492 have not been confirmed by subcloning and sequencing or an alternate methodology. Finally, some cases of disagreement appear difficult to explain on the basis of simple genotyping error or algorithmic bias, and may result from legitimate discordance in the underlying biological signal between the SNP and microsatellite assays (e.g., samples J249, L110). For example, some complex infections may contain highly related parasite strains that exhibit a polymorphic signal only in limited genomic regions that were differentially covered by these small panels of SNP and microsatellite markers.

**Figure 3:**
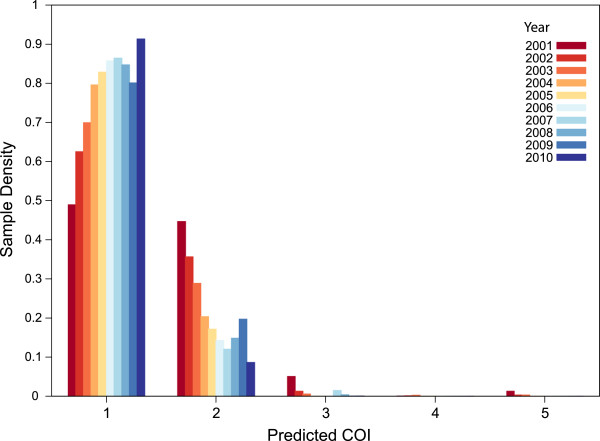
**Application to longitudinal genotype data** **(96 SNPs)** **from Thailand.** In concert with an observed decline in malaria transmission between 2001 and 2010 in Southeast Asia, COIL results indicate a steady shift in the COI distribution of malaria infections in a collection of 1,731 clinical samples. Data were previously published and obtained from [18].

Following these validation experiments, COIL was applied to several additional collections of patient samples. First, genotype data were acquired from a recently published collection of Illumina Goldengate genotyping results for 96 SNPs assayed in a longitudinal collection of 1,731 *P. falciparum* samples from northwest Thailand. The authors of the original study observed a steady decline from 2001-2010 in the proportion of samples exhibiting polymorphic SNP calls (63 *vs* 14% in 2001 *vs* 2010), and noted that this signal was among the strongest genomic correlates of the decrease in disease incidence and transmission observed in the region during that time interval. When these data are translated into COI estimates the decline in disease incidence manifests as a very consistent change in the distribution of COI in the patient population (Figure 3). Approximately half of the patient samples in 2001 exhibited COI >1, and over 5% exhibited COI >2. By 2010, over 90% of patient samples exhibited COI = 1, and virtually all complex infections were COI = 2.

**Figure 4:**
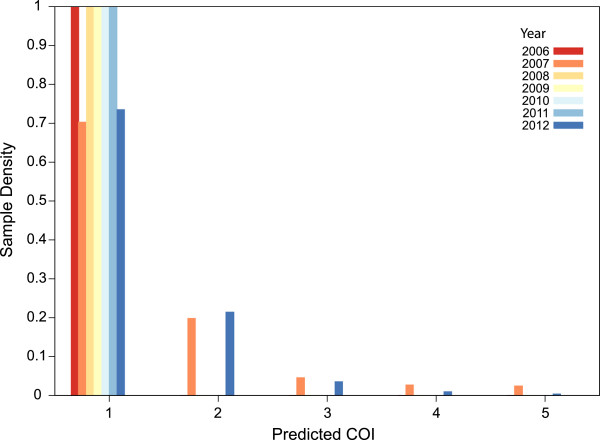
**Application to longitudinal genotype data** **(24 SNPs)** **from Senegal.** COIL reflects the dominance of single infections in this country for all years except 2007 and 2012, which exhibit infections with COI ranging up to five. Genotype data were collected from 974 clinical samples, some of which were analysed previously [26].

A comparable observation of longitudinal variation in the COI distribution over time can be made from SNP barcode data (Additional file 3) from *P. falciparum* samples collected in Senegal, using 24 SNPs genotyped with a high resolution melting (HRM) platform on 974 clinical samples collected between 2006 and 2012 [26], with MAF estimates derived from presumed singleton infections. The change in the distribution of COI exhibits less of a consistent temporal trend (Figure 4), possibly owing to lower baseline level of disease transmission. However, a clear decrease in the proportion of single infections is observable in 2007 and 2012 relative to other years, perhaps indicative of temporarily increased transmission rates during those intervals.

## Discussion

Two assumptions of independence were made in the creation of the COIL algorithm: 1) independence of genotypes among barcode assays, and 2) independence of parasite lineages within complex infections. To evaluate the usefulness of COIL in situations where one or both of these assumptions may be violated, the consequences of departures from these assumptions were evaluated. The binomial distribution is the basis of COIL, and is key to understanding the consequences of departures from the assumptions. The binomial distribution is the result of *n* independent Bernoulli trials, each of which return a binary outcome. This is applied in two ways to the data: first, whether a genotype call at a particular assay is polymorphic, and secondly, in the distribution of alleles at a binary assay. If *n* independent Bernoulli trials are performed each with the probability of success *p*, the expected value of the resulting binomial distribution is *np* and the variance is *np*(1-*p*).

Because Bernoulli trials are by definition independent, correlation between the trials naturally results in a different distribution. The expected value is the same, but the variance changes. If *X* is a random variable that is the sum of *n* Bernoulli random variables *X*_*i*_ each with probability of success *p*, where the *i*^th^ and *j*^th^ variables have correlation ρ _*ij*_, then the variance of *X* increases with ρ _*ij*_:


This equation illustrates that increased genotypic correlation will cause an increased variance, resulting in more extreme values in the correlated binomial distribution. This notion will be revisited below.

The first assumption is that the assays within a particular barcode are independent. This may not be true if a particular sample exhibits high levels of LD. The effects of this can be minimized by genotyping loci that are physically distant in the genome (at least 10-100 kb for most parasite populations [29]) or that reside on different chromosomes. In a highly inbred population, however, even polymorphic loci on different chromosomes may exhibit significant LD, making it impossible to conform with the assumption of assay independence.

Violation of the independent-assay assumption will result in overdispersion of the binomial distribution, and as a result COIL will render more extreme COI estimates (see *in silico* simulations in Additional file 4). If one member in a pair of correlated assays returns a polymorphic call, then the other member of the pair is likely to be polymorphic as well. The converse is also true in the case of monomorphic calls. Thus, an infection that has a true COI of three will be more likely to yield a COI estimate of two or four. The net result is that the COI estimates for individual samples will be less accurate. However, these antagonistic effects cancel each other in large collections of samples, and therefore population estimates of COI distribution are predicted to be unbiased by violation of the assumption of assay independence. This underscores the value of using large sample sets for obtaining accurate COI estimates with COIL.

COIL’s second assumption is that the individual parasite lineages comprising complex infections are randomly chosen from the larger parasite population, exhibiting a degree of genomic similarity to each other comparable to that observable between any set of strains observed in single infections. If this assumption is violated (for example if strains within complex infections exhibit higher genomic similarity to each other than randomly chosen strains from single infections) COIL will naturally underestimate the COI for such complex infections (Additional file 4). Recent common ancestry will result in a higher incidence of monomorphic genotypes, and there is no balancing effect as in the assay non-independence example.

Several studies suggest that complex infections may be serially co-transmitted, resulting in increasing levels of genomic homogenization via multiple generations of inbreeding [9, 32–34]. Over time, this process may yield complex infections composed of parasite strains exhibiting extremely high genomic similarity to each other. COIL is unequipped to reliably estimate COI in such situations, which may require single cell sequencing or genotyping approaches for precise disambiguation of highly similar genomic lineages.

A meristic measurement such as COI may be inappropriate for describing complex infections composed of highly similar parasite lineages, and that a continuous metric could better capture the complexity of such situations. In addition to discrete maximum *a posteriori* estimates, COIL provides a posterior distribution describing the uncertainty of the COI prediction, which may prove useful in detecting the presence of serially co-transmitted complex infections. The incidence of such infections and their relevance to predicting clinical outcome or understanding disease transmission dynamics remain to be thoroughly described, in part due to the current cost and technical complexity of single-cell genomic approaches.

This report demonstrates that the number of genetically unrelated parasite lineages in patient samples can be estimated inexpensively from SNP genotyping data, which can be derived from bulk genomic DNA extracted from a wide variety of clinical sample formats. The applications of such data for understanding disease etiology and epidemiology will multiply as the size and number of such datasets grow and yield new insights into parasite biology.

## Conclusions

COI will be a useful parameter to estimate to judge the efficacy of early-stage malaria elimination campaigns. This manuscript demonstrates that SNP genotype data have the potential to yield accurate estimates of COI, especially in cases where at least 96 SNPs are assayed and most SNPs exhibit a high MAF in the studied population. The COIL program is available for download from GitHub [1], and users may also upload their SNP genotype data to a web interface [2] for simple and efficient determination of sample COI.

## Electronic supplementary material

Additional file 1: **Microsatellite genotyping data for French Guiana parasite samples.** (XLSX 14 KB)

Additional file 2: **MAF data for French Guiana parasite samples.** (XLSX 49 KB)

Additional file 3: **SNP genotyping data for Senegal parasite samples.** (TXT 58 KB)

Additional file 4: **Simulations depicting the effects of violating assumptions made by COIL.** (PDF 153 KB)

## Abbreviations

COI: Complexity of infection
LD: Linkage disequilibrium
MAF: Minor allele frequency
PCR: Polymerase chain reaction
SNP: Single nucleotide polymorphism.
