# Supplementary material for: COIL: a methodology for evaluating malarial complexity of infection using likelihood from single nucleotide polymorphism data
Source: Malar J. 2015 Jan 19;14:4. doi: 10.1186/1475-2875-14-4 (PMC4417311; doi:10.1186/1475-2875-14-4)
Supplement: Supplementary file 4 — Additional file 4: Simulations depicting the effects of violating assumptions made by COIL. (PDF 153 KB) [file 12936_2014_3719_MOESM4_ESM.pdf]

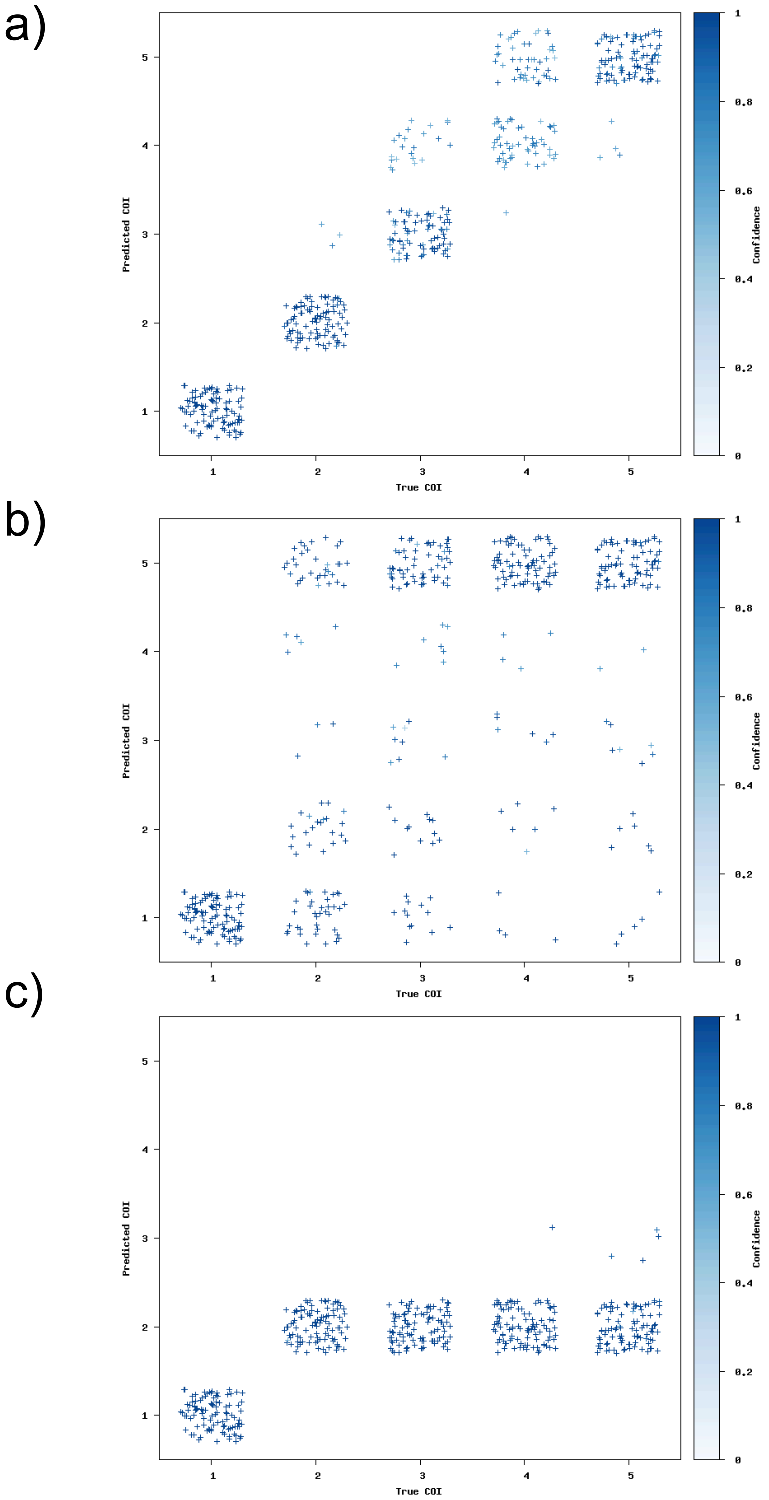

Additional file 4. Simulations depicting the effects of violating assumptions made by COIL. Horizontal axes represent true COI, vertical axes represent COIL maximum *a posteriori* estimates of COI. A total of 100 simulations were carried out for each COI level, with correlations between assays or strains defined as the probability that two non-independent Bernoulli trials yield a matching outcome. **a)** A simulation of 96 SNPs, MAF = 0.4, no correlation between SNP alleles or between strains occurring within mixed infections. **b)** A simulation of 96 SNPs, MAF = 0.4, correlation between SNP alleles = 0.4, no correlation between strains. COI predictions are skewed above or below the true COI. **c)** A simulation of 96 SNPs, MAF = 0.4, no correlation between SNP alleles, correlation between strains = 0.4. COI predictions are skewed below the true COI.
